# Invasive *Drosophila suzukii* facilitates *Drosophila melanogaster* infestation and sour rot outbreaks in the vineyards

**DOI:** 10.1098/rsos.170117

**Published:** 2017-03-29

**Authors:** A. Rombaut, R. Guilhot, A. Xuéreb, L. Benoit, M. P. Chapuis, P. Gibert, S. Fellous

**Affiliations:** 1 INRA, F-34988 Montferrier-sur-Lez, France; 2 CIRAD, UMR CBGP, F-34988 Montferrier-sur-Lez, France; 3 Université de Lyon, Université Claude Bernard Lyon 1, CNRS, Laboratoire de Biométrie et Biologie Evolutive, F-69100 Villeurbanne, France

**Keywords:** community ecology, *Drosophila suzukii*, *Drosophila melanogaster*, sour rot

## Abstract

How do invasive pests affect interactions between members of pre-existing agrosystems? The invasive pest *Drosophila suzukii* is suspected to be involved in the aetiology of sour rot, a grapevine disease that otherwise develops following *Drosophila melanogaster* infestation of wounded berries. We combined field observations with laboratory assays to disentangle the relative roles of both *Drosophila* in disease development. We observed the emergence of numerous *D. suzukii*, but no *D. melanogaster* flies, from bunches that started showing mild sour rot symptoms days after field collection. However, bunches that already showed severe rot symptoms in the field mostly contained *D. melanogaster*. In the laboratory, oviposition by *D. suzukii* triggered sour rot development. An independent assay showed the disease increased grape attractiveness to ovipositing *D. melanogaster* females. Our results suggest that in invaded vineyards, *D. suzukii* facilitates *D. melanogaster* infestation and, consequently, favours sour rot outbreaks. Rather than competing with close species, the invader subsequently permits their reproduction in otherwise non-accessible resources and may cause more frequent, or more extensive, disease outbreaks.

## 1. Introduction

*Drosophila suzukii* (Matsumara), the spotted wing drosophila, is an invasive Asian pest that was initially detected in Europe and North America in 2008 [1–3]. Unlike the vast majority of *Drosophila* species, which oviposit in decaying fruit, *D. suzukii* females can lay their eggs in undamaged, ripening fruit. *Drosophila suzukii*'s serrated ovipositor allows females to drill through fruit skin to access the flesh [4]. This invasive pest has a broad host range where larvae can develop in numerous wild and ornamental plants as well as cultivated berries and stone fruits [3,5]. Yield losses can reach 80% on crops such as strawberries, raspberries, blueberries and cherries [2,6]. Several biocontrol solutions including predators or parasitoids, fungal entomopathogens or nematodes have been tested, but yet none was successful [7]. Conventional pesticides are effective [8] but difficult to use, as fruit infestation occurs right before harvest at a time when most chemical treatments are prohibited for consumer health reasons [3].

*Drosophila suzukii* has only recently started to be seen as a threat to grapevines [9]. In 2014, wine producers in France and Western Europe lost up to 30% of their harvest due to sour rot [10], a grapevine disease that normally develops following *D. melanogaster* infestation of wounded berries [11]. The role of *D. suzukii* during this outbreak was suspected, as the invasive fly was captured in large number in fields with extensive sour rot cases [10]. However, a direct link between *D. suzukii* and the disease has not been established.

Sour rot is caused by the ongoing development of a community of acetic acid bacteria and yeasts [11]. The disease makes grapes unusable for both direct consumption and wine making [12]. Sour rot symptoms include the presence of acetic acid (giving a vinegar smell), produced by acetic acid bacteria of the genus *Acetobacter* sp. and *Gluconobacter* sp., and a characteristic grape colour change, where black grapes turn red and white grapes turn brown. While yeasts are not directly involved in the production of acetic acid, yeast species such as *Hanseniaspora uvarum*, *Pichia kluyveri* and *Candida stellata* are ubiquitous in sour rot-infected grapes [13].

Sour rot depends largely on Drosophilid infestation: after grapes are wounded by hail, birds or other insects, *D. melanogaster* and *D. simulans* can deposit their eggs onto exposed fruit flesh. *Drosophila* larvae are necessary for sour rot development because the movements of the larvae prevent the natural healing of small wounds [11,14]. It is possible that the bacterial and yeast species associated with sour rot are transferred to the open flesh by the flies, as these microbes are frequent on the cuticle and in the gut of *D. melanogaster* and *D. simulans* [14].

The role of *D. suzukii* in sour rot outbreaks is unknown [9]. A prevailing hypothesis is that fruit skin perforation by ovipositing *D. suzukii* females and larval activity initiate disease development. Bacteria and yeasts involved in sour rot disease are also found in wild *D. suzukii* flies where bacteria of the *Gluconobacter* and *Acetobacter* genera have been identified in the guts of wild adults [15] and the sour rot-involved yeast species *H. uvarum* and *P. kluyveri* were found in both adults and larvae [16].

To investigate the influence of *D. suzukii* on sour rot disease aetiology, we studied natural infestations in a vineyard where sour rot disease and *D. suzukii* were both present. We then conducted experimental infections in the laboratory to determine if *D. suzukii* can trigger disease development. Finally, we studied oviposition preference of *D. melanogaster* females in grape berries containing either *D. suzukii* or established sour rot in order to better understand the relationship between these two species and disease development.

## 2. Material and methods

All experiments and observations were conducted in a controlled climatic chamber with 14 h daylight per 24 h, 23°C and 75% RH.

### 2.1. Natural infestation in the field

This first experiment focused on the relationship between *D. suzukii*, *D. melanogaster* and sour rot in the field. We collected ripe grape bunches of the Meunier cultivar in Champagne vineyard (GPS coordinates 49.018, 3.982) where both *D. suzukii* and sour rot disease were present. We randomly sampled nine bunches without sour rot symptoms and nine bunches with severe sour rot symptoms (strong vinegar smell, typical reddish colour of the berries). Bunches were then individually placed in cages (cylinder of 25 cm diameter and 40 cm height, top covered with a 0.2 mm mesh net) where fly eggs, larvae and pupae present at the time of collection could develop, in the laboratory conditions described above. Adult flies emerging from the grapes were counted daily for two weeks and their sex and species identified. Eight days after collection, sour rot symptoms were re-evaluated. We determined three disease states of fruit degradation:

— No sour rot: the healthy-looking bunch at time of field collection still had no sour rot symptoms after one week.
— Post-harvest mild rot: the bunch was healthy when harvested, but showed symptoms of sour rot after one week.
— Severe rot: the bunch was harvested with severe sour rot that worsened for the duration of the experiment.

### 2.2. Presence of sour rot agents in wild *Drosophila suzukii* flies

As we collected grape bunches in the field, we also collected and isolated 15 wild *D. suzukii* flies from the same vineyard by net sweeping between plant rows. These wild flies were used to identify whole body-associated bacteria by high-throughput sequencing of a DNA amplicon coding for the 16S ribosome RNA gene. We used universal primers to amplify a 251-bp portion of the V4 region of the 16S rRNA gene (16S-V4F:587 GTGCCAGCMGCCGCGGTAA; 16S-V4R: GGACTACHVGGGTWTCTAATCC) and a dual-index method to multiplex our samples [17,18]. Laboratory preparation for DNA extraction, PCRs (in duplicate), and library preparation was performed as in [18].

With the pipeline FROGS [19], 2 × 251 bp paired-end sequencing data from the Illumina MiSeq system were processed. Briefly, we trimmed 3' primers with cutadapt [20], merged paired sequences into contigs with FLASH [21], filtered sequences by length (expected value of 251b ± 10b), dereplicated sequences, removed chimaeras using the algorithm of Edgar *et al.* [22] implemented in VSEARCH, clustered sequences with SWARM using a local clustering threshold [23] and returned taxonomic affiliation for each OTU using NCBI Blast + on Silva SSU 119 and 123.

Filtering for false positives was performed following the strategy proposed by Galan *et al*. [18]. In short, we discarded positive results associated with sequence counts below two OTU-specific thresholds, which controls respectively for cross-contamination (using six negative controls for extraction and PCR) and incorrect assignment due to the generation of mixed clusters during the sequencing (using four alien positive controls of either *Borrelia burgdorferi* or *Bartonella taylorii*). Finally, flies were considered positive for a given OTU only if both technical replicates were positive after the filtering steps.

### 2.3. Experimental infections

To determine whether *D. suzukii* can trigger sour rot, we exposed single grapes to one of four different treatments:

— with *D. suzukii*,
— with *D. melanogaster*,
— with both species, and
— with no fly.

Pristine single grape berries of the Red Muscat cultivar were isolated and checked for evidence of wounds, and the stems were still strongly attached therefore preventing access of flies to the berry flesh. In order to homogenize microbial communities on experimental fruits, we bleached berries in a 2.6% sodium hypochlorite solution and rinsed them in distilled water. We then dipped each berry in one out of four ‘sour rot extract’ which were produced by crushing four infected bunches collected in the infested vineyard for the previous experiment. Rot extracts were diluted 10 times in distilled water to reduce microbial inoculum concentration. Even if we kept track of rot extract identity, this factor had no influence on experiment outcomes and was discarded from statistical analyses.

Individual berries were placed in a cage (cylinder of 10.5 cm diameter and 8 cm height, top covered with a 0.2 mm mesh net) with either six *D. suzukii* females, six *D. melanogaster* females, six females from each species, or no fly. *Drosophila suzukii* flies were collected near Montpellier, southern France, in 2013 (PL-Mu strain). *Drosophila melanogaster* flies were the OregonR strain, founded in 1927 in the USA. Flies had been reared on banana medium for at least 2 years (electronic supplementary materials, table S1) and had never been exposed to grapes. They were not exposed to any specific treatment before the experiment and probably had similar bacterial community [24]. There were 17 single grape berries per treatment, each forming an experimental replicate.

After fly exposure for 72 h, the number of eggs laid by each *Drosophila* species was counted by visual inspection where egg species can be recognized by respiratory tube morphology. We monitored berries for sour rot symptoms appearance for one week. Another grapevine disease, the grey rot, (caused by *Botrytis cinerea*) developed on some fruits and was recorded. This fungus has a broad host range and a different aetiology to sour rot as it does not need wounds to develop [25]. It was present in two out of the four sour rot extracts mentioned above. Grey rot development was independent from fly treatment and was discarded from statistical analyses.

### 2.4. Behavioural assays

Two-choice tests were conducted with *D. melanogaster* females to determine whether they preferred laying eggs on wounded grapes infested with *D. suzukii* larvae or developing sour rot. We set up two experiments:

— choice between a sound grape and a grape inoculated with sour rot, and
— choice between a sound grape and a grape infested with *D. suzukii* larvae.

Danlas cultivar grapes were sanitized by washing in a 2.6% sodium hypochlorite solution and rinsed in distilled water. Grape berries were cut to expose 1 cm^2^ of flesh. We initiated sour rot disease by inoculating the wound with 500 µl of sour rot extract (see above). *Drosophila suzukii* infested grapes were obtained by transferring 10 *D. suzukii* eggs (PL-Mu strain) on exposed fruit flesh. Grapes were inspected until larvae emerged, and only grapes with at least five hatched larvae were used. Twenty-four hours after berry treatment, one *D. melanogaster* (OregonR strain) female was placed in a cage (cylinder of 10.5 cm diameter and 8 cm height, top covered with a 0.2 mm mesh net) with one of the two-choices tests. Females were removed the following day (*ca* 12 h later) and *D. melanogaster* eggs counted. Each treatment had 27 replicates, each involving a different *D. melanogaster* female.

### 2.5. Statistical methods

#### 2.5.1. Natural infestations in the field

We analysed separately the sums of *D. suzukii* and *D. melanogaster* adults that emerged from the grapes with generalized linear models. ‘Disease state’ was treated as a nominal factor, we specified a Poisson distribution and the log link function. We further used the least-square contrasts to determine differences between treatment levels.

#### 2.5.2. Experimental infections

We used a nominal logistic regression to analyse the proportion of fruits with sour rot or grey rot symptoms. We first analysed the effect of the exposure treatment (nominal factor). In a second stage, using only treatments with *D. suzukii* females, we analysed the effect of *D. suzukii* egg presence (i.e. whether or not we could observe eggs) on disease development. Pairwise test using Odds ratios were used to identify significant differences in disease prevalence among treatments levels.

#### 2.5.3. Behavioural assays

For each of the two-choice experiments, the number of *D. melanogaster* eggs deposited on each berry was analysed with a pairwise Wilcoxon's signed-rank test. All analyses were carried out within JMP Pro (SAS, 12.0.1).

## 3. Results

### 3.1. Natural infestation in the field

**Figure 1.**
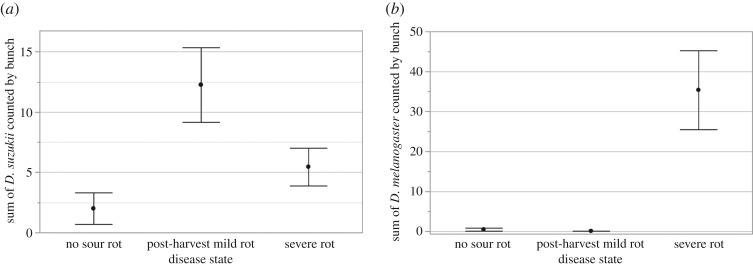
Emergences of (*a*) *D. suzukii* and (*b*) *D. melanogaster* from grapes collected in the field. ‘No sour rot’ indicates fruit without sour rot symptoms in the field and in the laboratory (*n* = 5 bunches), ‘Post-harvest mild rot’ indicates fruit that were collected without rot symptoms but showed mild rot in the laboratory one week after collection (*n* = 4 bunches), ‘Severe rot’ indicates fruit that already had severe rot symptoms when collected (*n* = 9 bunches). Black dots indicate means, error bars show standard error. Letters refer to significant differences calculated with contrasts. Note that the Y-scales differ among species.

In this first experiment, we monitored the emergence of *Drosophilae* from bunches collected in a vineyard in Champagne with both the presence of *D. suzukii* and sour rot. The number of emerging *D. suzukii* adults was significantly influenced by *Disease state* (*χ*^2^ = 10.02; *p* = 0.0067; figure 1*a*). More *D. suzukii* flies emerged from bunches with *Post-harvest mild rot* than with *No rot* or *Severe rot* (contrast: *χ*^2^ = 9.25; *p* = 0.0024).

The number of emerging *D. melanogaster* adults was significantly influenced by *Disease state* (*χ*^2^ = 30.60; *p* < 0.0001; figure 1*b*). *Drosophila melanogaster* almost only emerged from bunches with *Severe rot* (contrast: *χ*^2^ = 30.48; *p* < 0.0001).

### 3.2. Vectoring of sour rot agents by wild *Drosophila suzukii* flies

We also collected *D. suzukii* individuals in the Champagne vineyard to identify their bacterial communities using high-throughput sequencing. Overall, PCRs generated a minimum of 2000 reads per sample after filtering, for an average of 13 000 reads per sample, all the samples reached the plateau (see rarefaction curves in the electronic supplementary material, figure S1). Out of the 15 flies tested, we discarded the three which were positive for *Wolbachia* spp. because they yielded fewer than 2000 reads for other bacterial genera (for both amplification replicates). We assumed that the small number of sequences per sample would limit the completeness of bacterial detection. In the 12 remaining samples, we considered an OTU was present in a sample when the corresponding sequence was positive. All the samples contained acetic acid bacteria (table 1) and ten contained *Gluconobacter* spp. and *Acetobacter* spp., the most effective agents of sour rot [12].

**Table 1.** Presence of different types of bacteria in field captured *D. suzukii* adults as revealed by 16S meta-barcoding. Proportion of reads of a given bacteria taxon, prevalence among fly samples (*n* = 12 individuals), and number of OTU detected. Results are only detailed for the bacterial taxa involved in sour rot, the Acetobacteracae family, details for other families are presented in electronic supplementary material, table S2.

	*n* = 12 samples		
bacteria family	proportion of reads	prevalence	OTU
Acetobacteracae	0.438	12	9
* Acetobacter* spp.	0.031	10	1
* Commensalibacter* spp.	0.196	6	2
* Gluconobacter* spp.	0.052	10	3
* Komagataeibacter* spp.	0.158	9	3
other bacteria family (16)	0.561	12	26

### 3.3. Experimental infections

**Figure 2.**
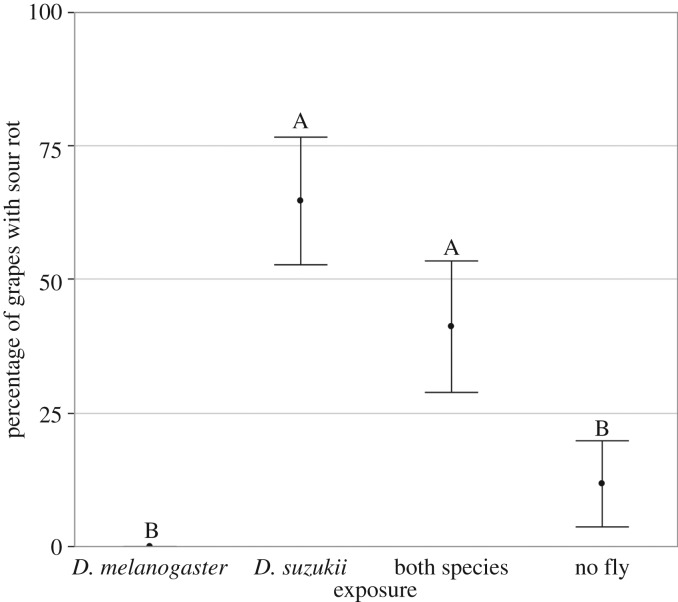
Effect of exposure to *D. melanogaster* and *D. suzukii* on sour rot onset in the laboratory. Percentage of berries that showed sour rot symptoms after exposure to different fly treatments (17 replicates per treatment). Black dots indicate means, error bars indicate standard error. Letters indicates significant differences among treatments.

We conducted trials to determine whether four exposure treatments (*D. suzukii, D. melanogaster*, both species or nothing) could trigger sour rot on a pristine grape berry. *Exposure treatment* had a significant effect on the presence of sour rot symptoms (*χ*^2^ = 24.96; *p* < 0.0001; figure 2), but not on grey rot development where 15% of grapes had grey rot distributed equally among treatments (*χ*^2^ = 0.47; *p* = 0.92). The two treatments with *D. suzukii* females (i.e. *D. suzukii* and *Both species*) induced sour rot symptoms more frequently than in the absence of *D. suzukii* (Odds ratios; *p* < 0.0039). When limiting the analysis to grapes exposed to *D. suzukii* females, all berries with eggs exhibited sour rot symptoms while only 15% of them did in the absence of visible eggs (*χ*^2^ = 21,23; *p* < 0.0001).

### 3.4. Behavioural assays

**Figure 3.**
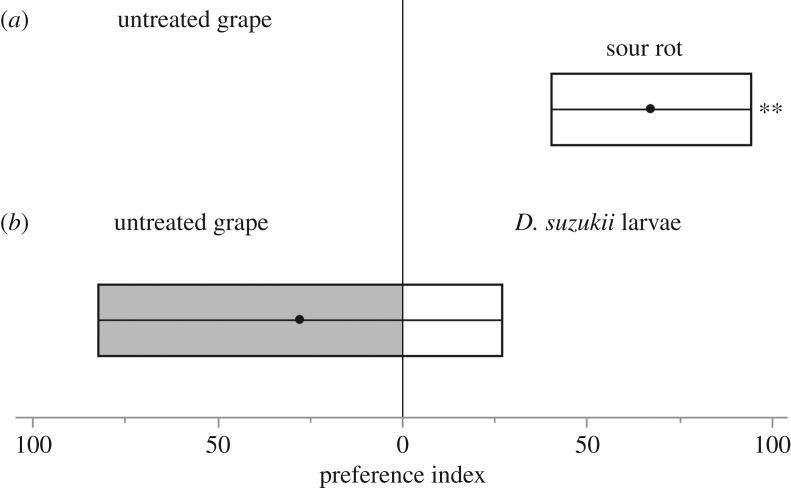
Oviposition preference of *D. melanogaster* female in 2-choices assays. Females had to choose between (*a*) untreated, wounded grapes and grapes with sour rot and (*b*) untreated, wounded grapes and grapes with *D. suzukii* larvae. For each experiment, there were 27 replicates each involving a different female fly. Black dots indicate means and boxes confidence intervals of estimated means. We used the following formula to represent the preference index: 100 × [no. of eggs on treated grape − no. of eggs on untreated grape]/total number of eggs. **p ≤ 0.01 from a pairwise Wilcoxon's signed-rank test.

Finally, we determined whether *D. suzukii* larvae and/or sour rot were attractive for *D. melanogaster* with two-choices oviposition assays. The presence of *D. suzukii* larvae on the grape had no significant effect on *D. melanogaster* oviposition preference (Wilcoxon-paired, *V* = 11.5, *p* > 0.4; figure 3*b*). However, sour rot disease significantly attracted ovipositing *D. melanogaster* females (Wilcoxon-paired, *V* = 109, *p* < 0.006; figure 3*a*).

## 4. Discussion

*Drosophila suzukii* emerged from grape bunches collected in the field more frequently when berries were healthy at the time of collection but showed mild symptoms of sour rot after one week in the laboratories (figure 1*a*). By contrast, numerous *D. melanogaster* emerged from bunches with severe sour rot symptoms in the field. Very few *D. melanogaster* flies emerged from bunches that appeared healthy when harvested, whether or not the symptoms were expressed later on (figure 1*b*). Meta-barcode analysis revealed that most *D. suzukii* adults from the field carried acetic acid bacteria (table 1). Experimental infections in the laboratories showed that exposure to *D. suzukii* females can trigger sour rot in healthy, intact fruit. Sour rot symptoms always appeared when *D. suzukii* females had laid eggs in the berry (figure 2). Finally, behavioural assays showed that ovipositing *D. melanogaster* females preferred sour rot-infected grapes, but not berries containing *D. suzukii* larvae (figure 3).

Our data confirm previous observations that *D. suzukii* is able to lay eggs in unwounded grape [4,9,10]. The high numbers of *D. suzukii* that emerged from bunches which developed sour rot in the laboratory (while looking healthy during collection) shows that *D. suzukii* and sour rot disease associate in the vineyard. Our laboratory experiments further confirmed that *D. suzukii* oviposition can trigger the onset of sour rot (figure 2), at least when acetic acid bacteria are present on the fruit surface as is common in the field [26]. In this assay, sour rot symptoms appeared in the presence of *D. suzukii* females and always occurred when *D. suzukii* eggs were observed (figure 2). Symptoms of sour rot when *D. suzukii* eggs were not observed are probably due to the difficulty of detecting deeply buried eggs.

The abundance of *D. melanogaster* adults that emerged from field bunches collected with rot symptoms contrasts with their absence from bunches that were healthy at the time of collection. This observation, in combination with results from experimental infections where *D. melanogaster* alone did not trigger sour rot symptoms on pristine grapes, is in agreement with previous publications that concluded the species cannot trigger the disease in unwounded grapes [11,12]. Before *D. suzukii* invaded French vineyards, wounds were due to climatic factors (for example, hail, heat shock, heavy rain leading to fruit skin shear or berry splitting) or physical damages by birds or wasps. As *D. suzukii* is present in the vineyard, these prior conditions seem no longer necessary as *D. suzukii* oviposition would suffice for disease onset.

Our behavioural assays results explain why high numbers of *D. melanogaster* flies emerged from bunches with severe sour rot symptoms. Ovipositing *D. melanogaster* females were neither attracted nor repelled by the presence of *D. suzukii* larvae (figure 3*b*). Sour rot, on the other hand, induced greater oviposition preference by *D. melanogaster* females (figure 3*a*). This was not surprising as this species of fly is known to be strongly attracted to acetic acid [27].

**Figure 4.**
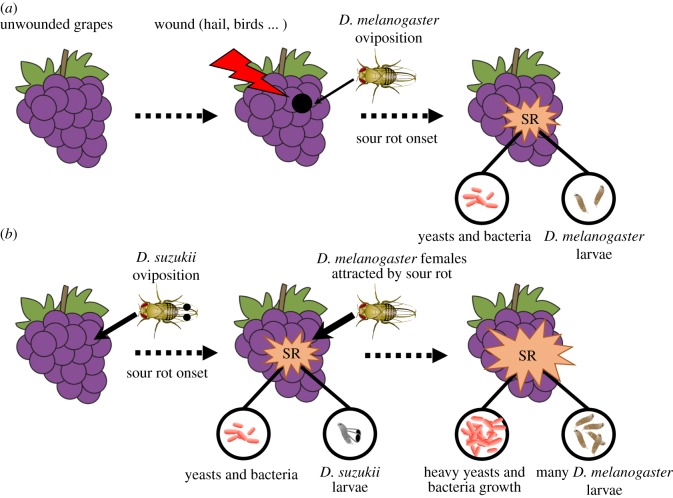
Scenarios of sour rot (SR) aetiology in the (*a*) absence and (*b*) presence of *D. suzukii*. Without *D. suzukii*, sour rot necessitates grape wounds in which *D. melanogaster* can lay its eggs. With *D. suzukii*, pest oviposition triggers sour rot onset, leading to earlier disease development and the production of odours that attract *D. melanogaster* females for oviposition. *D. suzukii* would hence facilitates *D. melanogaster* infestation and sour rot disease outbreaks. Note that spots on the wings of *D. suzukii* females and spotted wings on *D. suzukii* larvae were added for the figure's convenience.

By invading the vineyard agrosystems, *D. suzukii* facilitates both *D. melanogaster* reproduction and sour rot disease progression. Our results suggest that oviposition and larval development of *D. suzukii* induces sour rot disease in initially pristine grape berries, even in the absence of *D. melanogaster*. During early sour rot development, associated odours could attract *D. melanogaster* females that would also lay their eggs and colonize the fruit. This would lead to heavy infestations of *D. melanogaster* and large sour rot development (figure 4). The combined influence of *D. suzukii* and *D. melanogaster* would then increase the prevalence of sour rot disease in vineyards and worsen the damage it produces.

In most years, sour rot disease in European vineyards is sporadic where notable disease outbreaks depend on weather during grape ripening, high humidity levels and low temperature favouring the disease [28]. These climatic conditions are also favourable to the reproduction of *D. suzukii* [29,30]. This implies future outbreaks of sour rot might be more intense and more frequent than before *D. suzukii* invasion as favourable factors are similar.

In this study, we show for the first time that *D. suzukii* participates in the onset of a host plant disease. We also highlight how tripartite interactions between flies, plants and microorganisms are affected by the introduction of an invasive species, *D. suzukii*. The pest, by wounding the fruit during oviposition and inducing rot onset, allows the closely related species *D. melanogaster* to infest grape berries that would otherwise not be accessible. The invader therefore opens a new ecological niche for the native insect and its associated microbial community. Such positive effects of invasive alien species for native species are poorly documented; most studies focus on the negative effects of invasions on communities [31,32]. There are, however, other examples of facilitative effects of invasive species. For example, the non-native meadow *Lolium multiflorum* facilitates the native hemipteran pest *Stenotus rubrovittatus* in rice crops in Japan by being a host for the pest [33]. Gandhi & Herms [34] point out that invasive species might sometimes have positive effects on entire ecosystems, especially compared to the detrimental effects eradication methods could have. *D. suzukii* has a large spectrum of hosts, both cultivated and wild [5]. It is thus likely that invasion by this species has led to the spread of other wild plant diseases not yet documented and with unanticipated impacts on natural ecosystems.

## Supplementary Material

Table S1

## Supplementary Material

Figure S1

## Supplementary Material

Table S2

## Data accessibility

All the data will be available from the Dryad Digital Repository: http://dx.doi.org/10.5061/dryad.2jf75 [35].
