# Supplementary material for: Invasive Drosophila suzukii facilitates Drosophila melanogaster infestation and sour rot outbreaks in the vineyards
Source: R Soc Open Sci. 2017 Mar 29;4(3):170117. doi: 10.1098/rsos.170117 (PMC5383864; doi:10.1098/rsos.170117)
Supplement: Table S1 [file rsos170117supp1.docx]

| Ingredient | Volume |
| --- | --- |
| Distilled water | 1200 mL |
| Agar | 12 gr |
| Sugar | 74 gr |
| Dead yeast | 74 gr |
| Banana | 280 gr |
| Nipagine | 6 gr |
| Alcohol | 30 mL |

Table S1: Ingredient of the rearing medium of *D. suzukii* and *D. melanogaster* in the laboratory.
