# Supplementary material for: Invasive Drosophila suzukii facilitates Drosophila melanogaster infestation and sour rot outbreaks in the vineyards
Source: R Soc Open Sci. 2017 Mar 29;4(3):170117. doi: 10.1098/rsos.170117 (PMC5383864; doi:10.1098/rsos.170117)
Supplement: Figure S1 [file rsos170117supp2.docx]

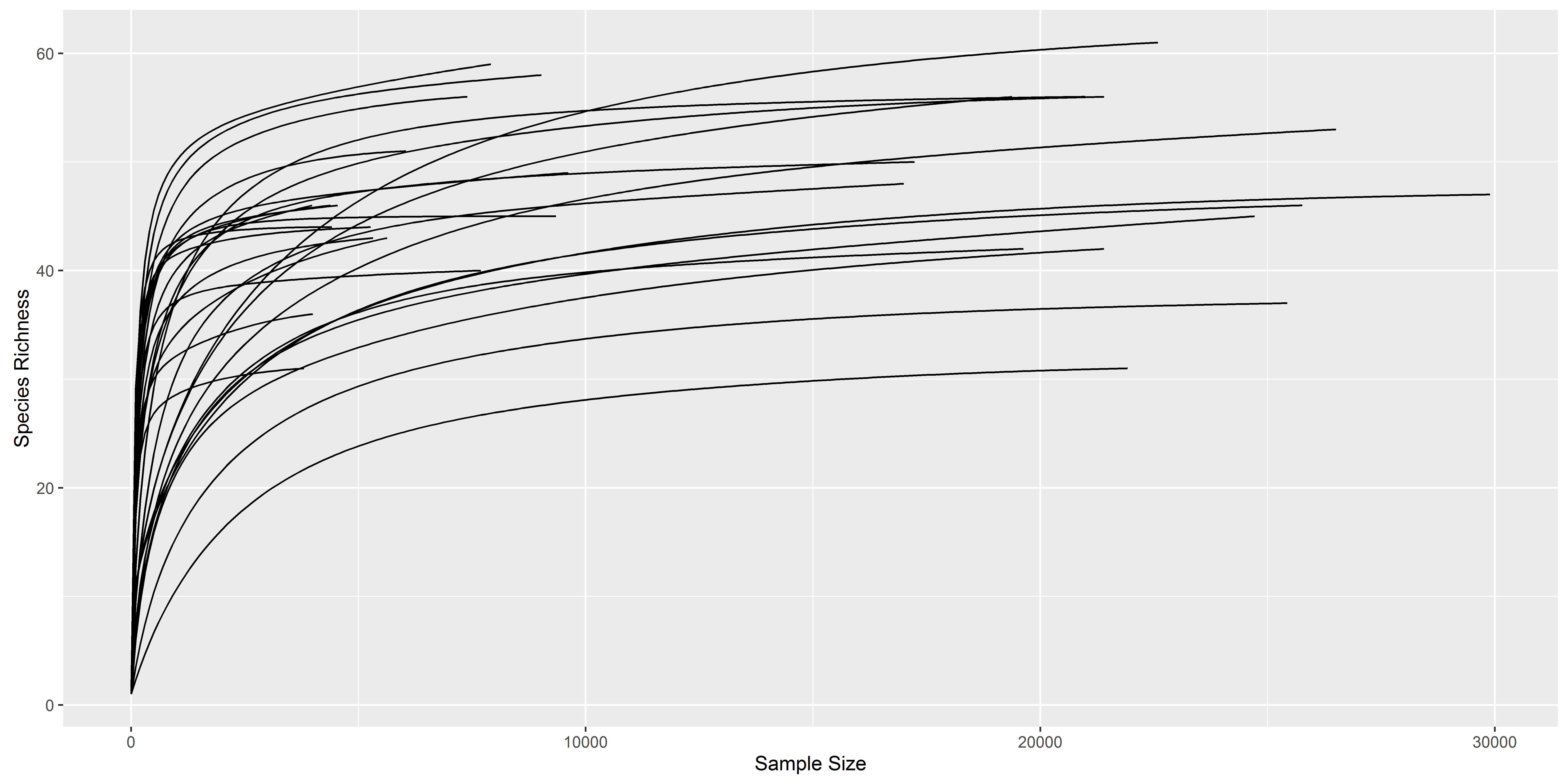


Figure S1: Rarefication curves of the 15 *D. suzukii* samples collected in Champagne (2 replicates by sample).
