# Supplementary material for: Invasive Drosophila suzukii facilitates Drosophila melanogaster infestation and sour rot outbreaks in the vineyards
Source: R Soc Open Sci. 2017 Mar 29;4(3):170117. doi: 10.1098/rsos.170117 (PMC5383864; doi:10.1098/rsos.170117)
Supplement: Table S2 [file rsos170117supp3.docx]

| Bacteria family |  | n=12 samples | | |
| --- | --- | --- | --- | --- |
|  |  | Proportion | Prevalence | OTU |
| Acetobacteracae |  | 0,438 | 12 | 9 |
| *Acetobacter* spp |  | 0,031 | 10 | 1 |
| *Commensalibacter* spp |  | 0,196 | 6 | 2 |
| *Gluconobacter* spp |  | 0,052 | 10 | 3 |
| *Komagataeibacter* spp |  | 0,158 | 9 | 3 |
| Anaplasmataceae |  | 0,089 | 6 | 1 |
| Burkholderiales |  | 0,023 | 12 | 2 |
| Chitinophagaceae |  | 0,003 | 1 | 1 |
| Enterobacteriaceae |  | 0,052 | 12 | 2 |
| Flavobacteriaceae |  | 0,032 | 9 | 2 |
| Gammaproteobacteria |  | 0,013 | 12 | 1 |
| Leuconostocaceae |  | 0,025 | 8 | 1 |
| Micrococcaceae |  | 0,059 | 12 | 4 |
| Moraxellaceae |  | 0,014 | 10 | 1 |
| Nannocystaceae |  | 0,003 | 3 | 1 |
| Neisseriaceae |  | 0,014 | 10 | 2 |
| Pseudomonadaceae |  | 0,059 | 11 | 2 |
| Sphingobacteriaceae |  | 0,044 | 8 | 2 |
| Staphylococcaceae |  | 0,02 | 12 | 1 |
| Xanthomonadaceae |  | 0,112 | 8 | 3 |

Table S2: Presence of different types of bacteria in field captured D. suzukii adults as revealed by 16s meta-barcoding. Proportion of reads of a given bacteria taxon, prevalence among fly samples (n=12 individuals), and number of OTU detected.
